# Deciphering tumor-infiltrating dendritic cells in the single-cell era

**DOI:** 10.1186/s40164-023-00459-2

**Published:** 2023-11-27

**Authors:** Qingyu Huang, Fuhao Wang, Di Hao, Xinyu Li, Xiaohui Li, Tianyu Lei, Jinbo Yue, Chao Liu

**Affiliations:** 1 Department of Radiation Oncology, Shandong Cancer Hospital and Institute, Shandong First Medical University and Shandong Academy of Medical Sciences, Jinan, 250117 China; 2 The Second Clinical Medical College, Anhui Medical University, Hefei, 230032 China; 3 Department of Oncology, Renmin Hospital of Wuhan University, Wuhan, 430060 China

**Keywords:** Dendritic cells, Single-cell sequencing technologies, Tumor microenvironment, Immunotherapy

## Abstract

Dendritic cells (DCs) serve as a pivotal link connecting innate and adaptive immunity by processing tumor-derived antigens and activating T cells. The advent of single-cell sequencing has revolutionized the categorization of DCs, enabling a high-resolution characterization of the previously unrecognized diversity of DC populations infiltrating the intricate tumor microenvironment (TME). The application of single-cell sequencing technologies has effectively elucidated the heterogeneity of DCs present in the tumor milieu, yielding invaluable insights into their subpopulation structures and functional diversity. This review provides a comprehensive summary of the current state of knowledge regarding DC subtypes in the TME, drawing from single-cell studies conducted across various human tumors. We focused on the categorization, functions, and interactions of distinct DC subsets, emphasizing their crucial roles in orchestrating tumor-related immune responses. Additionally, we delve into the potential implications of these findings for the identification of predictive biomarkers and therapeutic targets. Enhanced insight into the intricate interplay between DCs and the TME promises to advance our comprehension of tumor immunity and, in turn, pave the way for the development of more efficacious cancer immunotherapies.

## Introduction

The tumor microenvironment (TME) is a complex structure comprising immune cells, stromal cells, blood vessels, and extracellular matrix [1]. Immune cells play crucial roles in the TME and are typically categorized as adaptive and innate immune cells. Dendritic cells (DCs) are key intermediates proficient in antigen presentation, bridging the gap between innate and adaptive immunity [2]. DCs express receptors capable of recognizing diverse danger signals, including pathogens and altered cells, such as tumor cells [3]. Following antigen capture, activated DCs serve as specialized antigen-presenting cells [4]. They process both self and non-self antigens, subsequently presenting them to naïve T lymphocytes. These T lymphocytes, in turn, initiate antigen-specific immune responses while concurrently regulating tolerance and immunity [4].

The efficacy of the anti-tumor immune response relies on the cross-presentation of tumor-derived antigens by DCs to T cells, resulting in a predominant T cell-mediated cellular immune response [3, 5]. However, DCs infiltrating the TME display a heterogeneous nature characterized by variations in surface markers, migration patterns, localization, and cytokine production [6, 7]. Furthermore, distinct TME conditions can exert an influence on the effector functions of DCs, alter their phenotypic characteristics, and induce dysfunction and tolerance [8]. For example, studies have demonstrated that tumor-infiltrating DCs exhibit decreased expression of co-stimulatory molecules such as CD86 and CD80 [9], while concurrently displaying heightened expression of immune inhibitory molecules such as programmed cell death 1 ligand 1 (PD-L1) [10]. Therefore, comprehending the diversity of tumor-infiltrating DCs is crucial for the development of improved strategies for cancer immunotherapy.

Traditional bulk genomic and transcriptome analyses average signals across diverse cell groups, thereby hindering the identification of specific cell types and states [11]. Single-cell sequencing, however, offers the capacity to reveal transcriptomic cellular heterogeneity at a single-cell resolution, thereby exposing subpopulation structures that may remain indistinct in bulk RNA sequencing [11, 12]. The heterogeneity of DCs at the single-cell scale has been extensively explored in recent reviews [13]. In this context, we provide a succinct summary of DC subpopulations within the TME, elucidating their functions in various TME contexts, their roles in tumorigenesis and development, and their significance in ongoing anti-tumor therapies. This review centers on their tumor-related immune responses or pathways and their potential utility as predictive markers for therapeutic targeting.

### Advantages of single-cell sequencing

Cancer is characterized by its inherent heterogeneity and the complex composition of the TME [1]. Both tumor heterogeneity and the TME play crucial roles in tumor initiation, progression, invasion, metastasis, and drug resistance [14]. Bulk RNA sequencing technology primarily reveals an average gene expression profile within a sample, which poses challenges in comprehending tumor heterogeneity and the TME [11, 12]. The emergence and advent of single-cell sequencing technology have provided an opportunity to deconstruct the TME by discerning discrete cellular subpopulations, thus facilitating a more profound understanding of the intricate TME [11, 12]. In contrast to bulk sequencing, single-cell sequencing offers several distinct advantages, including its capacity to characterize cell subtypes and their relative frequencies within a sample, identify actively expressed genes within individual cells or cell types, and investigate communication between cells or cell types [15]. Recent advancements in single-cell sequencing techniques have undergone rapid development, with various applications and a primary focus on single-cell RNA sequencing (scRNA-seq). The critical determinant of success in single-cell studies lies in the preparation of high-quality single-cell suspensions. The process of single-cell suspension preparation encompasses density centrifugation for blood samples and mechanical enzymatic dissociation for solid tissues. Specific enzymes or mixtures are employed to facilitate effective cell separation, followed by DNase I treatment to minimize clumping. The choice of enzymes employed in various tumor models may exhibit slight variations depending on the tissue type. While Type IV collagenase is the standard choice in most scenarios, specific tissues such as the pancreas and intestine necessitate the utilization of alternative enzymes such as collagenase P and collagenase I [16]. In recent times, mixed enzyme products, such as Miltenyi Biotec's gentleMACS™ Dissociator, have become the preferred choice in the field and are frequently utilized in cancer studies for the preparation of single-cell suspensions [17]. This product exhibits effectiveness in dissociating tissues from various human and mouse tumor models, following meticulously designed procedures tailored to each tumor type. In summary, the acquisition of single-cell suspensions from diverse tumor models has evolved into a straightforward process. Subsequently, these suspensions are filtered through a mesh or strainer prior to single-cell capture. Short processing times are imperative to prevent gene expression variation and protect sensitive cells from damage. Alternatively, nuclear RNA sequencing is employed to alleviate biases stemming from cell type composition, particularly advantageous for intricate tissues such as interconnected adult neuronal tissues. This approach proves optimal for delicate cell types, such as differentiated neurons, providing valuable insights into their gene expression profiles, all without necessitating the isolation of intact cells [16]. In accordance with their experimental designs, researchers may find it necessary to augment or deplete specific cell types to increase the overall count of cells of interest in the final sequencing library. For instance, the analysis of specific immune responses may mandate the enrichment of immune cells, whereas cancer-related investigations may entail the exclusion of immune cells to boost the overall count of tumor cells.

Extensive transcriptomic information can be acquired through high-throughput scRNA-seq technology. Various downstream analysis tools facilitate the examination of both intra- and inter-tumor heterogeneity, mechanisms underlying tumor invasion and metastasis, TME characteristics, and the design of future treatment strategies. Corresponding bioinformatics methods have advanced to accommodate the complexities of scRNA-seq data, which are characterized by high dimensionality and the expression of numerous genes in each cell. Dimensionality reduction and clustering techniques empower researchers to categorize DCs into subpopulations with enhanced precision, thereby providing insights into the heterogeneity of traditional subtypes [18]. DCs exhibit intricate and diverse origins and developmental trajectories. Pseudotime trajectory analysis offers a means to elucidate the evolutionary progression of cells through gradual changes in gene expression. It can be employed to track cell lineage as well as to investigate the origins and differentiation of DCs [15]. Cellular communication through ligand–receptor interactions is linked to tumor progression in the TME [19]. Multiple analytical tools based on scRNA-seq data have the potential to reveal previously unexplored cellular receptor–ligand interactions critical for identifying prospective therapeutic targets [19]. The correlation among immune scores, prognosis, and responses to diverse treatments has been established [20, 21]. scRNA-seq provides an unprecedented level of resolution in characterizing infiltrating immune cells compared to conventional immune scoring methodologies, thereby enhancing the precision of prognosis and predictions for immunotherapy responses [22]. In addition, single-cell technology provides intricate details pertaining to individual cells across various dimensions. For example, the Cellular Indexing of Transcriptomes and Epitopes by Sequencing method enables simultaneous unbiased transcriptional profiling and antibody-based detection of protein markers in thousands of cells [23]. Single-cell analysis encompasses methylation patterns, histone modifications, chromatin accessibility, and T cell receptor repertoires, contributing valuable insights to cancer research from diverse perspectives [24–28]. The emergence of spatial transcriptomics allows for the simultaneous acquisition of cellular transcriptome data and information regarding cell locations, furnishing spatially informative datasets for TME investigations and addressing previous limitations in single-cell sequencing [11, 29, 30]. The TME consists of various cell types that frequently participate in well-organized spatial interactions [29, 30]. Deciphering this intricate spatial architecture enables us to grasp the mechanisms through which tumor cells communicate with each other, evade immune surveillance, and contribute to cancer progression. Therefore, investigating gene expression in a spatial framework offers a holistic comprehension of tumor initiation and facilitates the development of efficacious therapeutic strategies. These robust methodologies can assist in elucidating the heterogeneity of tumor-infiltrating DCs, thus offering comprehensive insights into cancer immunology research.

### Overview of DC subpopulations in human tumors

DCs represent a diverse group of immune cells, categorized into distinct subsets based on various criteria, including ontogeny, phenotypic characteristics, tissue distribution, and transcriptional profiles [6, 7]. DCs can be categorized into conventional or classical DCs (cDCs), which encompass type I cDCs (cDC1s) and type II cDCs (cDC2s), plasmacytoid DCs (pDCs), monocyte-derived DCs (moDCs), and LAMP3^+^ DCs. cDC1s excel in intracellular antigen processing and presentation, playing a crucial role in orchestrating anti-tumor immune responses. Their mechanism involves the cross-presentation of tumor-associated antigens to CD8^+^ T lymphocytes, which recognize these antigens through major histocompatibility complex (MHC) class I signaling [31]. Conversely, cDC2s efficiently present antigens associated with MHC II to CD4^+^ T cells, thereby promoting various T-helper (Th) cell responses, such as Th1, Th2, and Th17 cell polarization [31]. pDCs are major producers of type I interferons (IFNs) and are primarily involved in antiviral and antitumor immune responses [32]. moDCs represent a distinct subset that undergoes differentiation in response to inflammatory signals and is recruited to sites of inflammation, including the TME [33]. LAMP3^+^ DCs have been identified at the single-cell level and are distinguished by their immunoregulatory properties and migratory characteristics [10, 34].

**Fig. 1:**
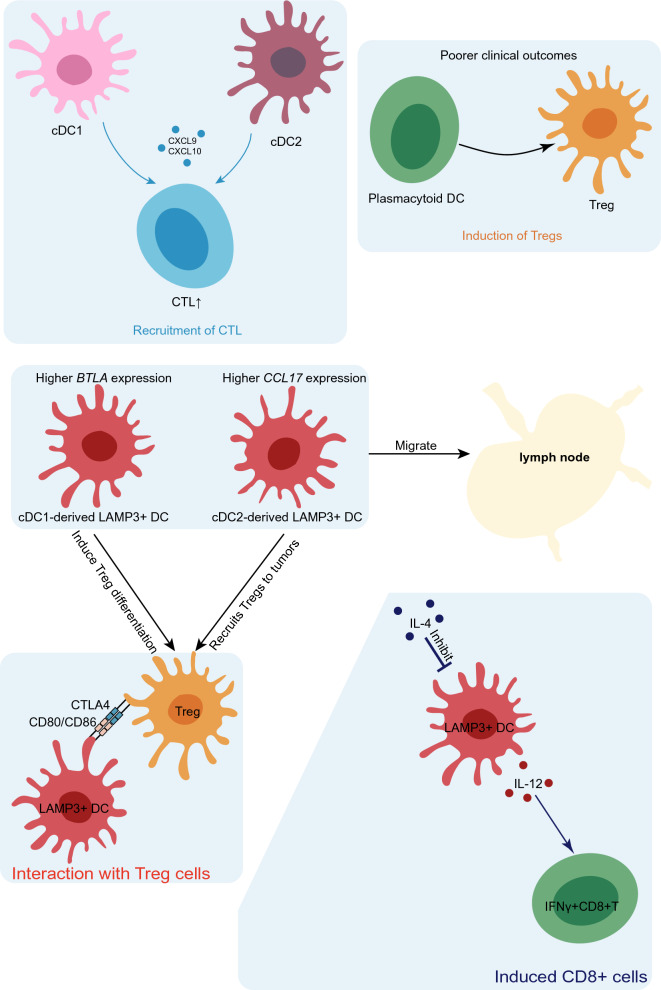
DC cross-talk within the TME. cDC1s and cDC2s recruit cytotoxic T lymphocytes (CTL) to the TME by secreting the chemokines CXCL9 and CXCL10, leading to an anti-tumor response. pDCs promoting the differentiation of Treg are associated with unfavorable clinical outcomes. LAMP3^+^ DCs, potentially originating from cDC1s and cDC2s, possess the capacity to migrate to lymph nodes. cDC1-derived LAMP3^+^ DCs notably express high levels of *BTLA* and drive Treg differentiation, whereas cDC2-derived LAMP3^+^ DCs exhibit elevated CCL17 expression, attracting Tregs to tumors. IL-12 expression in LAMP3^+^ DCs is downregulated by IL-4; however, blocking IL-4 augments IL-12 production and expands the population of IFNγ^+^ CD8^+^ T effector cells. LAMP3^+^ DCs inhibit T cell activation via CTLA4 and engage in interactions with Treg cells via CD80/CD86

Tumor-infiltrating DC states have been delineated through scRNA-seq across various human malignancies, encompassing breast cancer [35–37], hepatocellular carcinoma (HCC) [24, 34, 38, 39], colorectal cancer (CRC) [40, 41], non-small cell lung cancer (NSCLC) [9, 42–46], nasopharyngeal carcinoma (NPC) [47–49], esophageal squamous cell carcinoma [50, 51], glioma [52], cervical cancer [53, 54], gallbladder carcinoma [55], ovarian cancer [56–58], oral cancer [59], and gastric cancer (GC) [20, 60–63]. Pan-cancer analysis has indicated an enrichment of LAMP3^+^ DCs and pDCs in tumors, with both normal tissues and tumors demonstrating a comparable abundance of cDC2s and cDC1s. Among tumor tissues, cDC2s predominate [10, 64, 65]. The abundance of LAMP3^+^ DCs exhibits significant variability across different cancer types [10, 64, 65]. In various human malignancies, the transcriptional profiles and frequency of cDC1s are associated with improved survival rates and enhanced responsiveness to treatment [66, 67]. However, cDC2 exhibits heterogeneity, playing roles in both anti-tumor responses and tolerance processes within various TMEs [40, 68]. LAMP3^+^ DCs exhibit characteristics associated with both anti-tumor immunity and tolerance [10, 34]. In addition, the abundance and function of DCs display pronounced heterogeneity in the TME at different stages, underscoring their pivotal role in tumor immunity or tolerance [63, 69]. Hence, elucidating the biology of tumor-infiltrating DCs is critical for comprehending tumor immunity and advancing cancer immunotherapy. A deeper understanding of the complex TME via scRNA-seq will enable the identification of reliable predictive biomarkers and the development of novel therapeutic strategies. Here, we summarize the cell signaling/cell interaction (Table 1) and clinical relevance (Table 2) of tumor-infiltrating DCs and the mechanisms by which they interact with other cells in the TME (Fig. 1).

**Table 1:** Cell signaling/cell interactions in tumor-infiltrating DCs

Cell type	Cancer type	Cell signaling/cell interaction
cDC1	LUAD	TCF-1^+^ CD8 T cell reservoir [76]
	HCC	Elevated *HLA* gene expression and robust antigen presentation [39]
	TNBC	Activation of CD4-CXCL13 and CD8-CXCL13 T cells[35]
	GBM	Recruitment by intracellular cytotoxic T cells [94]
cDC2	LUAD	Reduced pro-inflammatory gene expression and increased anti-inflammatory signals [43]
pDC	LUAD	Upregulated expression of leukocyte immunoglobulin-like receptor genes, *granzyme B* production, and loss of *CD86, CD83, CD80*, and *LAMP3* markers [9]
	OSCC	Elevated *IRF8* levels, reduced IFN-α production, and promotion of Treg cell proliferation [82]
LAMP3^+^ DC	Pan-cancer	Elevated *BTLA/CCL17* expression inducing Treg differentiation [10]
	NSCLC	Enhancement of the population of IFNγ^+^ CD8^+^ T effector cells by IFN-γ-mediated IL-12 expression [85]
	NSCLC	Elevated PD-1 expression, upregulated by AXL receptor tyrosine kinase [85]
	NPC	Interaction with Treg cells via CTLA4 and CD80/CD86 [49]
	GC	Upregulation of the expression of IRF1, IRF2, NFKB1, and NFKB2 [84]
moDC	Peritoneal ascites from ovarian cancer patients	Antigen cross-presentation via vacuolar pathway and induction of cytotoxic CD8^+^ T cell differentiation [90]
	Thyroid cancer, glioma, and breast cancer	Released TSH promotes proliferation, invasion, and immune escape [52]

*cDC* conventional DC, *pDC* plasmacytoid DC, *moDC* monocyte-derived DC, *LUAD* lung adenocarcinoma, *HCC* hepatocellular carcinoma, *TNBC* triple-negative breast cancer, *GBM* glioblastoma, *OSCC* oral squamous cell carcinoma, *NSCLC* non-small cell lung cancer, *NPC* nasopharyngeal carcinoma, *TSH* thyroid-stimulating hormone, *GC* gastric cancer

**Table 2:** Clinical relevance of tumor-infiltrating DCs

Cell type	Cancer type	Clinical relevance
cDC1	LUAD	Contribution to anti-tumor immunity [76]
	TNBC	Positively associated with a favorable response to anti-PD-L1 therapy [35]
	Melanoma	Associated with improved clinical outcomes [77]
cDC2	Colorectal liver metastases	High proportion associated with poor prognosis [37]
pDC	NPC	Positively associated a favorable prognosis [45]
LAMP3^+^ DC	CRC	Early activation of primary myeloid cell type with anti-CD40 antibody treatment and association with overall survival [38]
	NSCLC	Positively associated with clinical response to neoadjuvant pembrolizumab and chemotherapy [44]
	CRC	Increased frequency after anti-CD40 antibody treatment and associated with favorable overall survival [41]
moDC	Melanoma	Upregulation of inducible iNOS, promotion of T-cell expansion, and anti-tumor immunity following combination treatment with anti-PD1 and anti-CD40 targeting moDCs [89]

*cDC* conventional DC, *pDC* plasmacytoid DC, *moDC* monocyte-derived DC, *LUAD* lung adenocarcinoma, *TNBC* triple-negative breast cancer, *NSCLC* non-small cell lung cancer, *NPC* nasopharyngeal carcinoma, *CRC* colorectal cancer, *iNOS* inducible nitric oxide synthase

### cDC1s

cDC1s, expressed *XCR1*, *CD8a*, *CLEC9A*, *CD103*, and *IRF8*, are essential for anti-tumor immunity [70]. cDC1s are employed for antigen cross-presentation and CD8^+^ T cell activation [71]. Experimental evidence suggests that tumor-infiltrating cDC1s promote tumor control in various ways, including the production of chemokines such as C-X-C motif chemokine ligand 9 (CXCL9) and C-X-C motif chemokine ligand 10 (CXCL10), which recruit CD8^+^ T cells to the tumor site. Additionally, cDC1s are involved in the local reactivation of these T cells via antigen cross-presentation and the production of various factors, such as IFN-λ [72]. The response-enhancing factors for cDC1s include type I IFN [73] and chemokines such as C–C motif chemokine ligand 5 (CCL5) [66], C–C motif chemokine ligand 4 (CCL4), and X-C motif chemokine ligand 1 (XCL1) [74, 75]. Moreover, intra-tumor NK cells play a pivotal role in the production of chemokines, such as CCL5 and XCL1 [66].

Single-cell sequencing studies have demonstrated the pivotal role of cDC1s in priming CD8^+^ T cells and orchestrating the CD8^+^ T cell response to checkpoint blockade immunotherapy [35]. The quantity and functional status of migratory cDC1s undergo alterations in response to tumor progression and can be strategically augmented to enhance tumor-specific CD8^+^ T cell responses [76]. Over time, the quantity and immune-boosting attributes of migratory cDC1s infiltrating the draining lymph nodes (dLN) decline, correlating with a diminishment in the anti-tumor CD8^+^ T cell response in the lung environment [76]. Employing a therapeutic approach to augment the migratory population of cDC1s by combining Fms-related tyrosine kinase 3 ligand with an agonistic CD40 antibody has been shown to promote SlamF6^+^TCF-1^+^ CD8 stimulation in dLN, subsequently resulting in enhanced T-cell trafficking into tumors and a reduction in tumor burden [76]. The cDC1s maintain a reservoir of proliferative tumor-antigen-specific TCF-1^+^CD8^+^ T cells in dLN [76]. Chuah et al. stratified patients with HCC who received anti-programmed death-1 (PD-1) immune checkpoint blockade into responders and non-responders and compared their DC populations [39]. They found that cDC1s, which were enriched in the responders, exhibited the highest expression levels of human leukocyte antigen (HLA) genes, indicating that cDC1s possess a significant capacity for antigen presentation [39]. Processes essential for immune priming, such as antigen presentation via MHC class II molecular processing, T-cell co-stimulation, and IFN-γ-mediated signaling, are all enriched in cDC1s [39]. Analysis of the temporal dynamics of different subsets of DCs in distinct treatment groups has revealed higher levels of cDC1s in responding patients who received a combination of paclitaxel and atezolizumab compared to those treated with paclitaxel alone, suggesting their involvement in anti- PD-L1 treatment [35]. Additional research has substantiated the association between cDC1s and a favorable response to combination therapy [35]. In summary, single-cell sequencing studies have yielded invaluable insights into the pivotal role of cDC1s in priming CD8^+^ T cells and improving responses to checkpoint blockade immunotherapy. These findings enhance our comprehension of the significance of cDC1s in antitumor immunity.

Numerous studies have demonstrated that cDC1s are essential for anti-tumor immunity. However, less is known regarding the signaling pathway governing cDC1's anti-tumor functionality in tumors [77]. Through single-cell transcriptomics analysis, Ghislat et al. investigated the molecular pathways regulating cDC1 maturation in tumors and found that the nuclear factor kappa-B (NF-κB) and IFN pathways are notably enriched in a subset of functionally mature cDC1s [77]. Experiments have demonstrated that the activation of the cDC1-related NF-κB/ Interferon regulatory factor 1 (IRF1) axis is associated with improved clinical outcomes in patients with melanoma [77]. Thus, the NF-κB/IRF1 axis in cDC1s may hold critical implications for the development of novel diagnostic and therapeutic strategies to improve cancer immunotherapy [77]. Single-cell communication analysis has enabled the identification of key pathways regulating anti-tumor resistance—a task that was previously unattainable through bulk RNA sequencing methods. In conclusion, the anti-tumor efficacy of cDC1s has been demonstrated and can be modulated through various mechanisms, such as cross-presentation and interactions with other cells in the TME. Exploring their functions and dissecting specific regulatory steps in the TME may offer novel avenues for advancing cancer therapy.

### cDC2s

The cDC2 subset, defined as *CD11b*^+^ *SIRPα*^+^ *CD1c*^+^, possesses the capability to activate CD4^+^ T cells. The cDC2 subset is now recognized as a heterogeneous population of cells, comprising true cDC2s and DC3s. scRNA-seq analysis has pinpointed DC3 as a specific subgroup sharing transcriptional characteristics with cDC2s and monocytes [78]. DC3s represent an inflammatory lineage of DCs with a notable capacity to modulate tumor immunity [78]. Both scRNA-seq data and batch gene expression profiles have elucidated shared marker genes between DC3s and cDC2s, such as *CLEC10A* and *FCER1A*, as well as between DC3s and monocytes, such as *S100A8*, *CD14*, and *CD163* [78]. DC3 and cDC2 exhibit numerous similarities, including the upregulation of cell surface co-stimulatory molecules such as CD80, CD86, and CD40, along with an increase in T cell-attracting chemokines [78]. Although DC3s can prime naive CD4^+^ and CD8^+^ T cells, they exhibit reduced efficiency compared to cDC2s. Nevertheless, DC3s excel at inducing a CD103^+^ tissue-resident phenotype in both CD4^+^ and CD8^+^ T cells.

Various studies have highlighted the heterogeneity of the cDC2 population. Brown et al. discovered that mouse cDC2s can be likened to the significant innate lymphoid cells or CD4^+^ T cell subgroup. It can be roughly categorized into two subgroups based on the presence or absence of T-bet (cDC2A and cDC2B, respectively), with the latter primarily expressing retinoic acid receptor-related orphan receptor γt (RORγt) [68]. Differential gene expression analysis of DC clusters has revealed that human cDC2A and cDC2B subgroups are characterized by mouse cDC2 subgroup-specific genes, similar to the mouse cDC2 differentiation pattern [68]. Human cDC2Bs demonstrate a more pro-inflammatory phenotype, while human cDC2As exhibit higher Amphiregulin levels [68]. Therefore, the unique expression patterns of T-bet and RORγt provide a foundation for the development of novel genetic tools, enabling more precise targeting of the cDC2 subpopulation and facilitating a deeper understanding of their roles in tissue homeostasis and immune regulation [68]. In CRC, four distinct cDC2 subgroups have been identified [40]. One subgroup exhibit "pro-inflammatory" characteristics, characterized by elevated gene expressions of *C1QA, CD68, CD163*, and *CD14*, while another subgroup exhibits high levels of "anti-inflammatory" gene expression, such as *CCR7*, as well as angiogenesis-related genes such as *EREG, CREM*, and *VEGFA* [40]. These genes play a critical role in angiogenesis and endothelial cell growth, promoting endothelial cell proliferation, cell migration, and vascular permeability [40]. The DC3 subtype has been identified as the predominant DC population in NSCLC and is enriched within NSCLC tumors [79]. Single-cell sequencing has revealed significant heterogeneity within the cDC2 population, enabling the identification of distinct subgroups with unique gene expression profiles, encompassing both pro-inflammatory and anti-inflammatory characteristics. This finer characterization of cDC2 subpopulations enhances our comprehension of their roles in tissue homeostasis, immune regulation, and the TME, providing valuable insights into potential therapeutic targets and strategies for cancer immunotherapy. However, our understanding of the composition, function, and differentiation of cDC2s and DC3s in the TME remains limited. Further investigation into cDC2 heterogeneity is crucial to understanding the mechanisms underlying TME immunosuppression or activation.

### pDCs

The pDC population exhibits elevated expressions of *LILRA4*, *CLEC4C*, *IRF7*, and *IL3RA* [36, 41]. In the TME, pDCs primarily secrete type I IFN, which can bolster antitumor immunity through interactions with both tumor and immune cells [32]. Chen et al. uncovered a significant association between the pDC signature and improved survival outcomes in NPC via scRNA-seq [47]. However, the question of whether pDCs are associated with a favorable or adverse tumor prognosis remains controversial. Previous bulk RNA sequencing studies have linked a higher proportion of pDCs to poorer clinical outcomes in ovarian cancer [57] and melanoma [80]. While pDCs display a diminished capacity for type I IFN generation, they exhibit an enhanced ability to induce Treg cell differentiation [57], potentially under the influence of transforming growth factor-β [81]. Furthermore, single-cell sequencing enables a focused exploration of the pDC subset, yielding more precise insights into its transcriptional characteristics. A single-cell study of lung adenocarcinoma revealed that pDCs exhibit an immunosuppressive phenotype characterized by the upregulation of genes from the leukocyte immunoglobulin-like receptor family, granzyme B production, and downregulation of activation markers, such as CD86, CD83, CD80, and LAMP3 [9]. In oral squamous cell carcinoma, pDCs express the highest levels of *IRF8* among various DC subsets, which is linked to their reduced ability to produce IFN-α [82]. Additionally, the analysis of pDC interactions with other immune cells has revealed their role in the TME. A significant correlation has been observed between exhausted CD8^+^ T cells and the increased abundance of pDCs in lung adenocarcinoma [83]. These findings underscore the significance of a personalized approach to modulating pDC function, offering the potential for targeted therapies aimed at optimizing anti-tumor immune responses across different contexts.

### LAMP3^+^ DCs

A recent study elucidated a subpopulation characterized by high *LAMP3* expression, which does not correspond to any canonical DCs in vivo. These LAMP3^+^ DCs express various maturation markers (*LAMP3*, *CD80*, and *CD83*), as well as migration markers (*CCR7*), along with lymphocyte recirculation chemokines (*CCL19* and *CCL21*) [34]. Zhang et al. utilized a migration score algorithm and determined that LAMP3^+^ DCs exhibit the highest migration score, suggesting that LAMP3^+^ DCs may represent the most activated DC subset, displaying potential migratory capacity within tumors. RNA velocity, an algorithm capable of inferring the evolutionary direction of subpopulations, indicates a directional evolution from cDC1s and cDC2s to LAMP3^+^ DCs [34]. The IRF family and NF-κB are pivotal regulators of DC differentiation and maturation [84]. Another study demonstrated that cDC1s and cDC2s enhance the expression of PD-L1, CD40, and interleukin-12 (IL-12) when encountering tumors, a pattern associated with the program of LAMP3^+^ DCs [85]. Additionally, cDC1s and cDC2s contribute to the population of LAMP3^+^ DCs in mice [85]. The mitochondrial phylogenetic tree of LAMP3^+^ DCs has revealed shared lineages in lymph nodes and tumors. RNA velocity analysis has demonstrated a directional flow of tumor-derived cells towards LAMP3^+^ DCs in LNs [34]. Collectively, LAMP3^+^ DCs may derive from cDC1s and cDC2s and have the ability to migrate to lymph nodes. LAMP3^+^ DCs from distinct sources exhibit varying functions (Fig. 1). cDC1-derived LAMP3^+^ DCs display elevated *BTLA* expression, which may induce Treg differentiation, thereby promoting immune tolerance [10]. In contrast, cDC2-derived LAMP3^+^ cDCs maintain high *CD1E* levels, a cDC2 marker gene, and demonstrate heightened *CCL17* expression. This chemokine recruits CCR4^+^ Tregs to tumor sites, creating an immunosuppressive environment [10].

LAMP3^+^ DCs demonstrate heightened immune activation in certain tumors. In CRC, the CCL22^+^ cDC1 subset has been identified as cDC1-derived LAMP3^+^ DCs. These CCL22^+^ cDC1s represent the principal myeloid cell type that undergoes early activation subsequent to anti-CD40 antibody treatment. Furthermore, the signature genes associated with activated cDC1s correlate positively with the overall survival of patients with CRC [41]. Activation of CCL22^+^ cDC1 cells following anti-CD40 treatment may elevate the occurrence of IFN-γ-producing tumor-infiltrating CD4^+^ Th cells [41]. Furthermore, this DC state has been found to be enriched in cases of microsatellite instability-high CRC [41], suggesting that CCL22^+^ cDC1s may serve as a valuable biomarker for assessing the efficacy of immunotherapy in CRC. In summary, single-cell sequencing technology empowers researchers to precisely identify and investigate relevant cell populations, thereby facilitating more in-depth research and potential therapeutic interventions. LAMP3^+^ DCs have also been associated with enhanced overall melanoma survival and a favorable modulation of the immune milieu through their influence on activated T cells and MHC expression [86]. Moreover, in NSCLC, IFN-γ upregulates IL-12 expression, whereas IL-4 downregulates it in LAMP3^+^ DCs; blocking IL-4 enhances IL-12 production and expands the population of IFN-γ^+^ CD8^+^ T effector cells [85]. LAMP3^+^ DCs also exhibit a high expression of PD-L1, with its upregulation being induced by the receptor tyrosine kinase AXL [85]. Hui et al. additionally observed that LAMP3^+^ DCs play a role in the recruitment and regulation of CD4^+^ T cells, CD8^+^ T cells, and B cells via multiple receptor–ligand interactions. This finding may have clinical relevance concerning the response to neoadjuvant pembrolizumab and chemotherapy in patients with NSCLC [44]. These studies suggest that LAMP3^+^ DCs interact with immune cells through diverse signaling pathways to activate the immune system and that targeting LAMP3^+^ DCs could potentially be an avenue to enhance antitumor immunity.

LAMP3^+^ DCs play a crucial role in the establishment of an immunosuppressive TME. In NPC, LAMP3^+^ DCs exhibit the highest levels of differentiation and apoptosis but the lowest levels of antigen presentation. They interact with Treg cells via Cytotoxic T-lymphocyte associated protein 4 (CTLA-4) and CD80/CD86, constituting a subset of regulatory and tolerogenic DCs that suppress T cell activation [49]. LAMP3^+^ DCs possess the capacity to modulate various T cell types via the PD-L1–PD-1 axis and are more likely to be associated with T cell dysfunction [34]. PD-L1 expression is universally upregulated in tumor-derived LAMP3^+^ DCs across nearly all cancer types [10]. CTLA-4 expressed on Treg cells exhibits a higher binding affinity for CD80 and CD86 on DCs compared to CD28, thereby limiting co-stimulatory signals and T cell activation [87]. PD-L1 also inhibits T cell proliferation and cytokine production by activating PD-1 [88]. These findings underscore the role of DCs in fostering tolerance in the TME. In conclusion, studies utilizing single-cell sequencing techniques have revealed LAMP3^+^ DCs as a distinctive and highly activated subset of DCs with migratory potential within tumors. They may originate from cDC1s and cDC2s and perform distinct functions contingent upon their source. Cellular communication analysis has also highlighted potential therapeutic targets. Consequently, deciphering the heterogeneity of LAMP3^+^ DCs and devising strategies to target them may provide new avenues for tumor therapy.

### moDCs

The moDC population exhibits elevated expression levels of *CLEC10A*, *HLA-DR*, *CST7,* and *CD1C* [89]. moDCs, originating from monocytes, are recruited to tissues and become the predominant DC population during inflammation [33]. Within the ascites of patients with ovarian cancer, a subset of moDCs capable of cross-presenting antigens via the vacuolar pathway and inducing cytotoxic CD8^+^ T cell differentiation has been identified [90]. The CD28 co-stimulatory T cell receptor represents the primary target of PD-1-mediated inhibition [91]. A recent study revealed that moDCs in the TME exhibit heightened CD86 expression, which may be implicated in CD28-dependent PD1 blockade. This heightened CD86 expression positively correlates with the abundance of effector tumor-infiltrating lymphocytes under conditions of successful anti-PD-1 treatment [89]. The combination of anti-PD-1 and anti-CD40 immunotherapy results in the upregulation of inducible nitric oxide synthase (iNOS) in moDCs, thereby facilitating T cell expansion and promoting anti-tumor immunity (Fig. 1), suggesting that moDCs could serve as a valuable therapeutic target for combination therapy [89]. In the TME of thyroid cancer, moDCs displaying high levels of *TSHα* and *TSHβ2* expression represent the primary source of thyroid-stimulating hormone (TSH). TSH released by moDCs promotes the proliferation, invasion, and immune evasion of TSH receptor-high tumors, such as thyroid cancer and glioma [52]. In summary, despite the limited scRNA-seq data available on moDCs, these cells are known to exert a significant influence on shaping the TME and hold promise as potential therapeutic targets. Their impact on immune responses, particularly in the context of combination therapies, underscores the need for further investigation of moDCs through single-cell sequencing techniques.

### Pan-cancer analysis of DC subsets

According to the clustering of identical cDC subsets, the transcriptome status of cDC subsets exhibits remarkable consistency across diverse cancer types [10]. However, the regulatory factors of PD-L1 in LAMP3^+^ DCs are intricate and display considerable variation among different cancers, despite its upregulation being a common feature in nearly all cancer types [10]. Although DCs exhibit a conserved profile, a pan-cancer analysis revealed heterogeneity among tumors. Notably, the proportion of cDC2s exceeds that of cDC1s in tumors, and the abundance of LAMP3^+^ DCs varies significantly across different cancer types [10]. However, another pan-cancer analysis revealed that cDC2s are the most abundant DC subset, with the numbers of other DCs varying according to the specific cancer type [65]. cDC2s have been found to be enriched in migratory branches, and migratory DCs may originate from cDC2s as opposed to cDC1s in tumors [65]. Hong et al. discovered that LAMP3^+^ DCs and pDCs are enriched in tumors, whereas both normal tissues and tumors exhibit comparable levels of cDC2 enrichment [64]. Conversely, cDC1s predominate in normal tissues adjacent to tumors or in normal donors but are rare in blood or lymph nodes [64]. The cDC2 proportion exhibits substantial variability among patients, and the expression of marker genes associated with cDC2 positively correlates with the survival of patients with cancer [64]. Tregs and cDC2s are primarily involved in mediating communication between DCs and T cells [64].

### DC signatures of tumorigenesis and progression

**Fig. 2:**
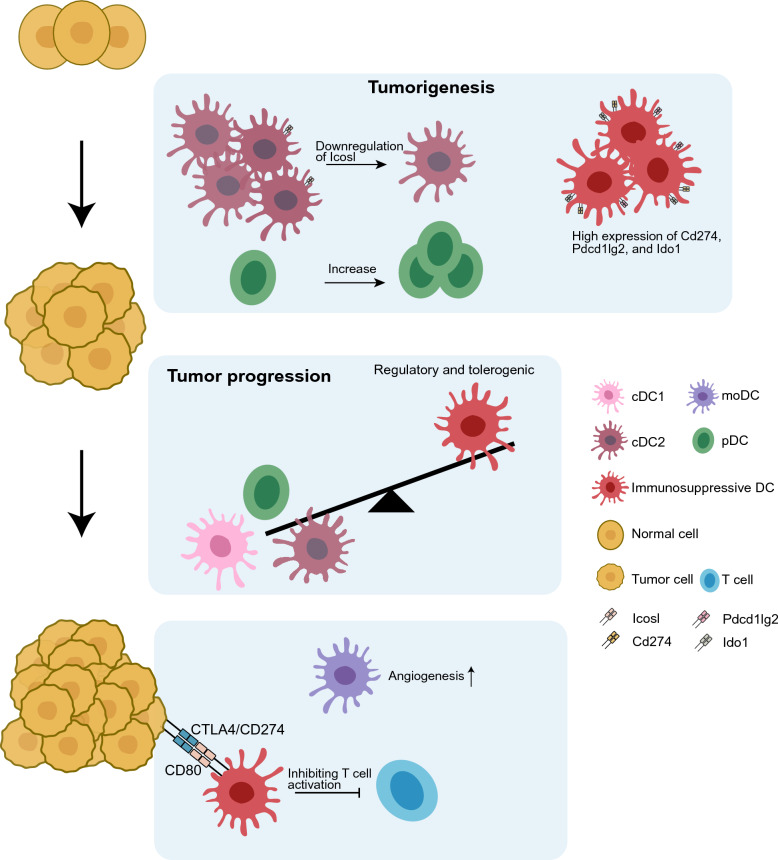
DC signatures of tumorigenesis and progression. The proportion of pDCs increases during tumorigenesis, accompanied by a decrease in cDC2s and a downregulation of ICOSL. LAMP3^+^ DCs exhibit elevated expressions of immune checkpoint molecules CD274, PDCD1LG2, and IDO1. As tumors progress, the abundance of cDC1/2 and pDCs decreases, while LAMP3^+^ DCs become more abundant. In recurrent or metastatic tumors, LAMP3^+^ DCs interact with tumor cells via CD274/CD80 and CTLA4/CD80, suppressing T-cell activation. moDCs exhibit a signature associated with angiogenesis. Various DC subpopulations are present at different stages of tumors. The figure illustrates subpopulations that undergo significant quantitative or functional changes during tumor development

Extensive research focused on the functions of DCs in tumor development and progression has been conducted, emphasizing the significant alterations in DC populations and functions across various cancer types. A comprehensive understanding of the impact of these changes on immune responses may facilitate the development of targeted and efficacious therapeutic strategies. Figure 2 illustrates the characteristics of DCs during tumorigenesis and progression. In cervical cancer, the abundance of cDC2s decreases as the cancer progresses [54]. Additionally, cDC2s exhibit decreased expression of pro-inflammatory genes and increased levels of anti-inflammatory signatures along the spatial continuum from normal tissue to lung adenocarcinoma [43]. Gallbladder carcinoma-induced tumorigenesis creates an immunosuppressive environment characterized by an increase in pDCs [55]. Conversely, during esophageal carcinogenesis, the proportion of cDC2 decreases, and the expression of inducible T cell costimulator ligand (*ICOSL*), a gene indicative of immunostimulatory activity, is downregulated [69]. Immunosuppressive DCs characterized by elevated expressions of PD-L1, programmed cell death 1 ligand 2 (PD-L2), and indoleamine 2,3-dioxygenase 1 (IDO1) are consistently observed throughout tumorigenesis, in contrast to cDC2s, which exhibit reduced immunostimulatory activity [69]. pDCs are predominantly detected in primary GC and lymph node metastases, while their presence is rare in paracancerous tissues [62]. Recurrent NPC exhibits a higher frequency of LAMP3^+^ DCs, whereas primary NPCs are enriched in cDC1/2 and pDCs [48]. Moreover, in recurrent NPC, DCs upregulate tolerogenic pathways such as IL-18 production, leading to T cell tolerance induction and the inhibition of T cell activation, thereby demonstrating enhanced regulatory and tolerance mechanisms [48]. DC3s are more prevalent in primary tumors than in liver metastases and non-metastatic CRC tumors. In contrast, cDC2-TIMP1 exhibits the highest rate of liver metastases [40]. Primary and metastatic GC show enrichment in cDC1s and cDC2s, while paracancerous tissues exhibit a higher proportion of activated DCs [62]. Furthermore, in response to anti-tumor immunity, cancer cells can develop mechanisms to exploit DCs, thereby promoting immune tolerance. Recurrent HCC tumor cells interact with cDC2/LAMP3^+^ DCs via CD274/CD80 and CTLA4/CD80, leading to the formation of clusters around DCs and subsequently reducing antigen presentation and T cell activation efficiency [38]. Monocyte-like DCs found in ascites in patients with advanced GC exhibit diminished antigen presentation and increased pro-angiogenesis, accompanied by heightened expression of immune checkpoint genes and a more unfavorable prognosis compared to early-stage GC [63]. A study investigating cervical carcinogenesis revealed a significant accumulation of pDCs in cervical cancer as the disease progresses [92]. This accumulation is accompanied by reduced IFN-α production, elevated immunosuppressive mediator expressions (*IDO1* and *PD-L1*), and mitogen-activated protein kinase and NF-ĸB pathway enrichment. These findings suggest a potential oncogenic role for pDCs during chronic viral infections [92]. In conclusion, DCs are likely to play a pivotal role in tumor development, and gaining a deeper understanding of their functions could contribute to the development of more effective therapeutic strategies.

### DCs and antitumor therapy

The role of DCs in antitumor therapy varies depending on the specific cancer type and treatment approach (Table 3). In preclinical studies, the presence of IL-12-producing tumor-infiltrating DCs proves to be indispensable for the antitumor response [93]. IFN-γ and IL-12-mediated communication between T cells and DCs activates antitumor T cell immunity [93]. Currently, the methods based on single-cell technology employed to investigate DCs and antitumor therapy primarily involve the analysis of alterations in the quantity or functionality of DC subpopulations before and after treatment, or to assess the effects of different treatment modalities on DC subpopulations.

**Table 3:** DCs and antitumor therapy

Treatment	Cancer type	Findings
Anti-PD-L1 therapy	Breast cancer	Increased cDC1, LAMP3^+^ DC, and pDC levels are associated with therapeutic response [35]
Chemotherapy	Gastric cancer	Number of DCs decreases around tumors after two cycles of chemotherapy [61]
Neoadjuvant immunochemotherapy	NSCLC	Significantly higher expression of LAMP3 after neoadjuvant immunochemotherapy [44]
Neoadjuvant PD-1 blockade combined with chemotherapy	NSCLC	Increased proportion of cDC1s and cDC2s in responding patients and an accompanying increase in antigen presentation characteristics [45]
Neoadjuvant chemotherapy	Esophageal adenocarcinoma	cDC suppression in the TME was corrected, and pDCs were significantly reduced [95]
Neoadjuvant chemoradiotherapy	Esophageal squamous cell carcinoma	Decrease in cDC1s and LAMP3^+^ cDCs [51]
Neoadjuvant anti-PD1 therapy	Recurrent glioblastoma	Specific chemokine receptor–ligand interaction between XCR1 and XCL1/2, suggesting the recruitment of cDC1 by intratumoral cytotoxic T cells [94]
Immunotherapy	Gastric cancer	Proportion of moDC clusters decreases compared to chemotherapy, antigen presentation and pro-angiogenic capacity downregulated, and an anti-inflammatory phenotype in response to immunotherapy [63]
Radiochemotherapy	Cervical cancer	Decreased cDC1 relative proportion, increased gene expressions associated with leukocyte migration and activation, and enrichment of antigen processing and presentation [53]

*cDC* conventional DC, *pDC* plasmacytoid DC, *TME* tumor microenvironment, *moDC* monocyte-derived DC, *NSCLC* non-small cell lung cancer

**Fig. 3:**
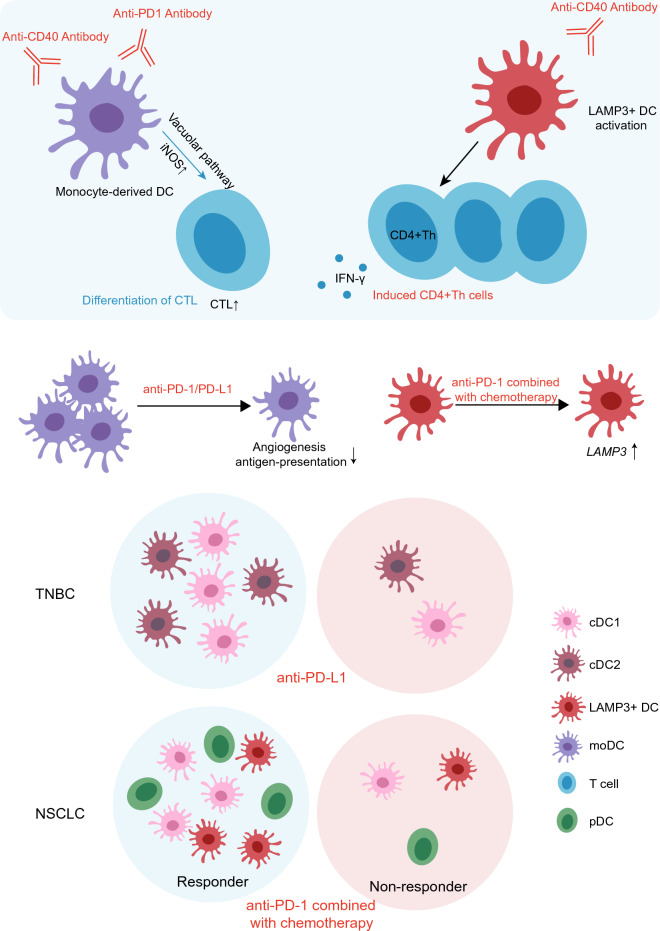
DCs and immunotherapy. Combining anti-PD1 and anti-CD40 immunotherapies enhances inducible nitric oxide synthase (iNOS) expression in moDCs, thereby promoting the differentiation of cytotoxic T lymphocytes (CTLs). Anti-CD40-induced activation of LAMP3^+^ DCs may augment the frequency of IFN-γ-producing CD4^+^ Th cells within the TME. Following anti-PD-1/PD-L1 treatment, a reduction in the proportion of moDC clusters occurs, leading to diminished antigen presentation and pro-angiogenic capabilities. The significant upregulation of LAMP3 expression following neoadjuvant immunochemotherapy indicates their active involvement in treatment response. In NSCLC, elevated levels of cDC1, LAMP3^+^ DCs, and pDCs are associated with treatment response. In triple-negative breast cancer, an increased proportion of cDC1 and cDC2 is linked to treatment response

DCs play a crucial role as antigen-presenting cells in cancer immunotherapy. Here, we summarize the role DCs play in immunotherapy (Fig. 3). Patients with breast cancer who respond to anti-PD-L1 therapy display elevated levels of cDC1s, LAMP3^+^ DCs, and pDCs, suggesting their potential contribution to the therapeutic response [35]. Moreover, the suppression of LAMP3^+^ DCs has demonstrated the association between these DCs and the effectiveness of PD-L1 blockade following paclitaxel treatment in combination with anti-PD-L1 therapy [35]. Furthermore, LAMP3 expression levels are significantly higher in patients with NSCLC who undergo neoadjuvant immunochemotherapy compared to untreated patients, underscoring the involvement of LAMP3^+^ DCs in actively regulating the therapeutic response to neoadjuvant immunochemotherapy [44]. Another study involving patients with NSCLC who received a combination of neoadjuvant PD-1 blockade and chemotherapy revealed that patients who respond positively exhibit an increased proportion of both cDC1s and cDC2s, coinciding with an enhancement in their antigen presentation capabilities [45]. Furthermore, a specific chemokine receptor–ligand interaction has been observed between XCR1 (cDC1) and XCL1/2 (predominantly expressed by cytotoxic T cells) in patients with recurrent glioblastoma (GBM) who receive neo-adjuvant anti-PD-1 therapy, suggesting the recruitment of cDC1s by intratumoral cytotoxic T cells [94]. However, this phenomenon has not been observed in patients newly diagnosed with GBM or in those with recurrent GBM who had not previously undergone immunotherapy [94]. Immunotherapy has been shown to reduce the proportion of moDC clusters compared to chemotherapy in GC [63]. The downregulation of antigen presentation and pro-angiogenic capacity in response to immunotherapy has also been observed in moDC clusters, signifying an anti-inflammatory phenotype in response to immunotherapy [63]. Studies have examined the impact of radiotherapy and chemotherapy on the abundance and function of DCs during treatment. In patients with GC, those who experience rapid disease progression following chemotherapy initially exhibit a high abundance of CD11C^+^ DCs surrounding the tumor. However, these DCs nearly disappear after two cycles of chemotherapy [61]. Conversely, in patients with GC who are unresponsive to chemotherapy, the number of DCs surrounding tumors significantly decreases after two cycles of chemotherapy [61]. Neoadjuvant chemotherapy corrects the suppression of cDCs in the TME and results in a significant reduction in pDCs in patients with esophageal adenocarcinoma who receive neoadjuvant chemotherapy [95]. Additionally, cDC1s and LAMP3^+^ DCs decrease following neoadjuvant chemoradiotherapy in esophageal squamous cell carcinoma [51]. After radiochemotherapy in cervical cancer, a decrease in the relative proportion of cDC2s, an increase in gene expressions associated with leukocyte migration and activation, and an enrichment in antigen processing and presentation have been observed [53]. These findings suggest that the function of DCs in antitumor therapy may be influenced by the type of cancer and the specific treatment strategy employed. Current research has primarily focused on monitoring alterations in DC count caused by treatments, with the underlying mechanisms remaining obscure. Elucidating the interactions between distinct DC subtypes and antitumor therapies will enhance our comprehension of tumor elimination.

### DC-based tumor immunotherapy

Immunotherapy has demonstrated clinical benefits across various cancer types. Various preclinical and clinical investigations have employed DC-based tumor immunotherapy [96]. The DC vaccine is a safe and effective means of inducing tumor immunity. Personalized DC vaccines targeting tumor-specific antigens have shown promise in inducing T-cell immunity among patients with melanoma [97]. In patients with HER2-positive breast cancer, DC vaccines induce specific tumor-specific T-cell responses [98]. Moreover, the DC vaccine, in combination with other immunotherapies, has yielded compelling results in several studies. In a mouse model of head and neck squamous cell carcinoma, the DC vaccine enhanced the antitumor effect of anti-PD-L1 monoclonal antibodies [99]. The combination of the DC vaccine and ipilimumab has produced notably high and sustained tumor response rates in patients with advanced melanoma [100]. Recent clinical trials in the realm of DC-related cancer immunotherapy have primarily focused on DC vaccines, while potential therapeutic targets identified through single-cell sequencing are still pending validation.

## Conclusions

Single-cell sequencing has revolutionized our understanding and comprehension of the TME and tumor-infiltrating DCs. Single-cell sequencing techniques have enabled the identification of reliable predictive biomarkers and novel therapeutic approaches for cancer treatment by offering high-resolution categorization of DC subgroups and characterizing their heterogeneity. Additionally, the capability to simultaneously profile multiple cellular attributes at single-cell resolution has provided unprecedented insights into intercellular communication within the TME, which is closely linked to tumor progression. These investigations have shed light on the heterogeneity and functional versatility of DCs across various cancer types. However, several essential questions and limitations require further exploration.

The diversity and plasticity of DCs in tumors remain incompletely elucidated. Despite the identification of distinct DC subsets, an ongoing debate persists regarding their precise origins, functions, and interactions within the TME. It is essential to acknowledge that the classification of DC subpopulations can vary across studies, posing challenges to the establishment of a universal consensus. Moreover, the roles of distinct DC subsets in tumor immunity are complex and can vary across diverse cancer types and stages. In addition, the impact of DCs on antitumor therapies is multifaceted, with various treatment modalities, including immunotherapy, chemotherapy, and radiotherapy, potentially impacting the abundance and function of DCs within the TME. Leveraging single-cell technology allows us to capture a momentary snapshot of the TME at a specific time point. We recognize, however, that this approach may not fully encapsulate the dynamic changes that transpire within the TME over time. It is imperative to acknowledge that the TME is susceptible to temporal fluctuations and responds variably to therapeutic interventions. Currently, studies concerning the TME landscape at various time points remain limited in their ability to fully elucidate the dynamic alterations of DCs, potentially attributable to the challenges associated with multi-time point sampling. Therefore, it will be imperative, in the future, to expand the scope of analyses to encompass multiple time points, thereby affording a more comprehensive understanding of how DC populations evolve in response to disease progression and various treatment modalities. Furthermore, DC-based tumor immunotherapy, particularly DC vaccines, holds promise in the realm of cancer treatment. Understanding the mechanisms by which distinct DC subtypes interact with these treatments and the ensuing impact on tumor immunity is essential for the optimization of therapeutic strategies. However, the translation of these findings into effective clinical therapies necessitates further validation and refinement. Clinical trials exploring the potential of DC-based immunotherapies are still ongoing.

In conclusion, single-cell studies have provided valuable insights into the intricate landscape of DCs in human tumors. These studies have underscored the potential of DCs as therapeutic targets and as indicators of treatment responses. Nonetheless, additional research is required to elucidate the functional complexity of DC subpopulations, their interactions with other immune cells, and their roles in the dynamic TME. Such knowledge is critical for the development of more effective cancer therapies and for advancing our understanding of tumor immunity.

## Data Availability

Not applicable.

## Abbreviations

DC: Dendritic cell
TME: Tumor microenvironment
PD-L1: Programmed cell death 1 ligand 1
PD-1: Programmed death-1
scRNA-seq: Single-cell RNA sequencing
cDC: Conventional DC
pDC: Plasmacytoid DC
moDC: Monocyte-derived DCs
cDC1: Type 1 conventional DC
cDC2: Type 2 conventional DC
MHC: Major histocompatibility complex
Th: T-helper
IFN: Interferon
HCC: Hepatocellular carcinoma
CRC: Colorectal cancer
NSCLC: Non–small cell lung cancer
NPC: Nasopharyngeal carcinoma
GC: Gastric cancer
dLN: Draining lymph nodes
CRC: Colorectal cancer
GBM: Glioblastoma
HLA: Human leukocyte antigen
CXCL9: C-X-C motif chemokine ligand 9
CXCL10: C-X-C motif chemokine ligand 10
CCL5: C–C motif chemokine ligand 5
XCL1: X-C motif chemokine ligand 1
NF-κB: Nuclear factor kappa-B
IRF1: Interferon regulatory factor 1
RORγt: Retinoic acid receptor-related orphan receptor γt
CTLA-4: Cytotoxic T-lymphocyte associated protein 4
PD-L2: Programmed cell death 1 ligand 2
IDO1: Indoleamine 2,3-dioxygenase 1
iNOS: Inducible nitric oxide synthase
TSH: Thyroid-stimulating hormone
